# Contamination of Homes with Methamphetamine: Is Wipe Sampling Adequate to Determine Risk?

**DOI:** 10.3390/ijerph16193568

**Published:** 2019-09-24

**Authors:** Jackie Wright, G. Stewart Walker, Kirstin E. Ross

**Affiliations:** 1 College of Science and Engineering, Flinders University, Adelaide 5042, Australia; jackie.wright@flinders.edu.au (J.W.); stewart.walker@flinders.edu.au (G.S.W.); 2 Environmental Risk Sciences, Sydney 2118, Australia

**Keywords:** methamphetamine, risk, exposure, house, home, domestic dwelling

## Abstract

Contamination of domestic dwellings from methamphetamine cooking or smoking is an increasing public health problem in many countries. To evaluate the extent of contamination, sampling generally focusses on the collection of surface wipe samples from walls and other surfaces of a potentially contaminated home. Here, we report the contamination levels of many household materials and items sampled from a home that was suspected to be the premises used to cook methamphetamine, it was then sold, lived in for several years by the new owners and then left unattended for several more years. Although the time since the cooking had taken place was significant (over five years), the levels of contamination were extremely high in both household items that were part of the house when cooking was taking place (blinds, carpets, walls, etc.) and also in articles brought to the house post-cooking (rugs, toys, beds, etc.). Both wipe sampling and analysis of bulk samples indicate that the methamphetamine is not breaking down or being removed and is transferred from contaminated to non-contaminated objects. These results raise questions about the adequacy of characterising contamination and of making decisions about the extent of remediation required based solely on surface wipe samples. Without fully understanding the extent of contamination that is present, not only on surfaces but within the building materials, it is difficult to ensure that the correct and most effective remedial approaches are taken to appropriately determine and address the risks to inhabitants.

## 1. Introduction

Methamphetamine contamination of domestic dwellings resulting either from cooking or smoking methamphetamine is an increasing problem in many countries [1,2]. Most cooking is undertaken in domestic dwellings [3]. Unless a property has been seized by police, there is often no evidence of the home having been used to smoke or cook methamphetamine, and renters or purchasers of properties are often unaware that their home is contaminated [4]. Non-users can be affected by exposure as a result of subsequent contact with high levels of illicit drug residues on surfaces in the home environment (third-hand exposure) [5]. To protect public health, guidelines for indoor surface residues for residential and commercial premises are available [6,7]. In Australia, investigation levels for methamphetamine are 0.5 µg methamphetamine per 100 cm^2^ and 10 µg/100 cm^2^ for residential and commercial properties, respectively [8]. These guideline values act as triggers for both assessment and validation for remediation.

While it is assumed that contamination from methamphetamine smoking is likely to be less than that arising from methamphetamine manufacturing in homes, research showed that levels of 1.5–5 µg/100 cm^2^ could be reached from smoking if multiple smoking sessions were undertaken (based on experimental data designed to simulate multiple smoking sessions or a multiple-user session) [9]. Data from former clandestine drug laboratories in Australia [10] found that around 50% of those clan labs tested had levels up to 10 µg/100 cm^2^, with the rest reporting levels as high as 4000 µg/100 cm^2^.

The assessment of methamphetamine contamination inside a home is assessed (in Australia) in summary by the Australian Crime Commission [8] as:

(a) Taking at least five surface wipe samples from within the premises.
(b) Taking surface wipe samples from areas that show evidence of contamination, surfaces used in the drug manufacturing process and any room inhabited by a child less than 16 years of age.

These guidelines are similar to recommendations published elsewhere [11,12,13].

However, there is evidence to suggest that the concentrations on different surfaces differ markedly [14,15], as does the transferability [16]. This could have significant implications for assessing properties’ potential risk to public health and for assessors establishing that risk through direct measurements.

Here we report the levels of contamination of methamphetamine on different surfaces and in materials from a dwelling that was suspected to have been used to cook methamphetamine. The details of how the property came to be owned by the current owners, the levels of exposure and the health impacts associated with exposure in this house have been reported elsewhere [4]. Briefly, the property was suspected to have been used to cook methamphetamine during an unknown time, and in May 2013, police seized chemicals and manufacturing equipment from a shed on the rural property. While the house was not listed on the police report as a location of suspected manufacture, subsequent testing indicates that manufacture is likely to have occurred throughout the property. The property was not assessed nor remediated prior to sale. The house was unwittingly purchased in August 2013 and was occupied by the family from October 2013. During this time, the occupants did not undertake any renovations, including painting, as the house was recently constructed. The family moved out in March 2015 and the house remained unoccupied. During this time the house was closed, windows were shut and no remediation took place. Most of the samples reported here were collected in April 2017.

As part of the assessment of potential methamphetamine contamination at this property, wipe sampling was undertaken in the home by various testing companies at different times. The wipe sampling was undertaken using standard wipe sampling methods, with samples mainly collected from the painted plasterboard walls. These data were used to determine contamination levels and inform the selection of appropriate remediation methods.

Further testing was subsequently undertaken in this home to obtain more data about the level of methamphetamine present on and within common household items and also within various building materials. The purpose of collecting this additional data was to understand how much methamphetamine was held within the various building materials and furnishings, how much methamphetamine was transferred to the family possessions and whether this information differed from that provided by standard surface wipe testing.

## 2. Materials and Methods

### 2.1. Surface Wipe Sampling

Some surface wipe sampling of internal painted gyprock walls of the house was undertaken by commercial testing companies for the purpose of assessing the contamination status and determining appropriate remedial options. Additional surface wipe samples from the internal walls of the home were taken at various times to further document any changes in contamination levels over time.

The testing was completed by a remediation company in October 2014; the primary author of this article (hereafter J.W.) in March 2017 and by J.W. in November 2018. Surface wipe samples were collected using a technique compliant with NIOSH 9111. This involves a clean cotton gauze swab pre-moistened with 3 mL methanol (or isopropyl alcohol). The sample is collected from a 100 cm^2^ template area and collected as follows:

(a) Wipe the surface to be sampled with firm pressure using vertical S-strokes. Fold the exposed side of the pad in and wipe the area with horizontal S-strokes. Fold the pad once more and wipe the area again with vertical S-strokes (i.e., the area is wiped 3 times).
(b) Fold the pad exposed side in, place in a 25 mL container for shipping and seal with a cap.

Wipe samples were not taken from the same 100 cm^2^ area at each sampling event. One other sampling event was undertaken by another company but was not compliant with NIOSH methodology, so that data are not reported here.

### 2.2. House Material Sampling

**Figure 1:**
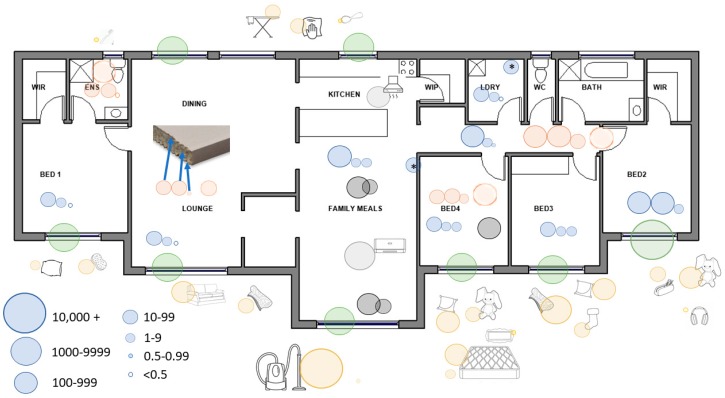
Distribution of methamphetamine throughout the family home. Key: blue = walls [L to R: outer paper (that facing the room), gyprock centre, inside paper (that in contact with the wooden structure)], green = blinds, orange = ceiling (L to R: outer paper, gyprock centre, inside paper), orange with blurred edge = insulation above the ceiling, yellow = toys etc (household goods brought into the house after purchase), grey = carpets and underlay, pale grey = filters taken from the air-conditioner and the kitchen extractor. Units = µg/100 cm^2^ (or estimated equivalent).

House materials were sampled over the period of one day in April 2017. Materials sampled were those that were present during the time of suspected methamphetamine cooking which included roof insulation, plasterboard (wall pieces), timber frame, carpet, blinds and filters from the rangehood in the kitchen area and from the air conditioner in the living area of the open-plan room as shown in Figure 1.

Materials were also sampled from items that were brought into the property by the family at some stage after suspected manufacture, including rugs, mattresses, personal items, toys and cooking implements. The contents of the vacuum cleaner bag and an unused vacuum cleaner bag were also sampled.

Samples were individually sealed in clean sample bags and stored within the same storage room at Flinders University, Adelaide, South Australia, for approximately 16 months prior to analysis. No other materials were stored in this storeroom that contained or were contaminated with methamphetamine. The samples remained sealed to prevent contamination by the external environment and to prevent the escape of contaminants within the samples.

### 2.3. Analyses

All samples were shipped to commercial laboratories under the chain of custody protocols for analysis for methamphetamine, amphetamine, pseudoephedrine and ephedrine.

#### 2.3.1. Surface Wipes

The surface wipe samples collected were all analysed by commercial laboratories. Laboratory analysis of samples by JW was via a modified NIOSH 9111 method (extraction using 0.1 M sulfuric acid and analysis by LCMSMS). Samples were analysed using a modified NIOSH 9106 method (solvent extraction and analysis by LC–MS (Shimadzu LCMS 8060 with a C18 column, Shimadzu, Kyoto, Japan)). The analytical limit of reporting (LOR) for these analyses was 0.02 µg/sample.

#### 2.3.2. House Materials

The bulk samples collected from the house were analysed using a modified NIOSH 9111 method. It should be noted that the analytical method is validated only for the analysis of surface wipe samples and has not been validated for all the different matrices collected (currently there are no laboratories that have validated methods for the analysis of the range of matrices sampled). As a consequence, the results obtained from the analysis of bulk samples (i.e., not the wipe samples) can be considered as indicative only.

Many of the samples provided to the laboratory were large items. These samples were sub-sampled by the laboratory for analysis. For these items a section of approximately 100 cm^2^ was sub-sampled. For large items such as curtains and plasterboard, an approximate 100 cm^2^ sample was analysed.

For larger personal items, the 100 cm^2^ subsample was made up of a number of smaller samples taken from all over the item.

All samples of approximately 100 cm^2^ in size were weighed and then digested in accordance with the analytical method, with 1 g of the digested sample analysed. The mass of chemical detected in the whole sample was extrapolated from this analysis to the mass of the sample. This provided a mass detected in the sample of approximately 100 cm^2^ in size. For a comparison with surface wipe samples, the results were reported as μg/100 cm^2^ (even when that is not technically possible). For accuracy, the bulk analysis result reported as μg/g was also provided.

To determine the profile of methamphetamine contamination through wall material, the plasterboard was separated into three samples, including the paper on either side and the internal plaster. Each component was analysed separately.

For the analysis of items that could not be tested as bulk samples, a wipe sample was collected from the surface of the item. Where possible, the wipe sample was collected over a 100 cm^2^ area with sampling and analysis as described above.

For the samples, the analytical limit of reporting (LOR) was 0.2 µg/sample. However, for the results reported as μg/g, the LOR may be presented as <0.04 μg/g. For these samples, methamphetamine (or other analyte) was detected (as indicated by the mass detected in µg) but at a level that did not reach the LOR applied for the reporting of bulk analysis results. Owing to these constraints, values are reported for two significant figures.

## 3. Results

Surface wipe samples collected from the internal painted gyprock walls of the home were not always collected from the same rooms during every sampling event. However, methamphetamine was detected in all samples collected at levels ranging from 0.54–110 µg/100 cm^2^, with an average level from all samples analysed at 31 µg/100 cm^2^ as shown in Table 1. All levels of methamphetamine reported in the home exceeded the ACC guideline of 0.5 µg/100 cm^2^. Amphetamine was also detected in most samples. The precursor chemicals, pseudoephedrine and ephedrine (also present as impurities in the manufactured drug), were only detected in some samples, particularly those where higher levels of methamphetamine were reported.

In relation to the house materials samples, methamphetamine was detected in almost all the samples analysed as shown in Figure 1. Amphetamine was also detected in most samples analysed. The precursor chemicals, pseudoephedrine and ephedrine, were detected in a number of samples including the ceiling insulation, blinds (curtains), outside paper (the paper exposed to the room) of the plasterboard samples, vacuum cleaner bag and in other samples where higher levels of methamphetamine were reported (data not shown).

There are no guidelines available for bulk analysis of building materials that determine a safe level that can be present as μg/g (i.e., the mass of the drug/chemical in the mass of the item sampled). The current residential guidelines from the ACC [8] relate to the mass that may be present on surfaces (over 100 cm^2^) that may then be transferred to and absorbed via dermal absorption or incidental ingestion by children and adults. Here, we present estimates of mass over the surface area to allow comparative estimates of contamination.

For the materials that were present in the property prior to occupancy, methamphetamine concentrations are considered to be significantly elevated. This includes roof/ceiling insulation (0.61–4.1 μg/g or 56–430 µg), blinds (15–150 μg/g or 2200–20,000 µg), carpets (0.23–3.7 μg/g or 30–370 µg), filters from the rangehood and air conditioner (21–36 μg/g or 530–3600 µg) and the plasterboard (with elevated levels in the outer paper up to 170 μg/g or 700 µg; with lower levels in the gyprock material and inside paper) as shown in Figure 1. The wipe sample collected from the timber frame piece detected methamphetamine at 1.1 µg/100 cm^2^.

Materials that were brought into the property when occupied sometime after the suspected manufacture are also contaminated, with elevated concentrations of methamphetamine reported. This includes rugs (0.93–17 μg/g or 42–5100 µg), mattresses (15 μg/g or 180 µg on the outer layer and 0.09 μg/g or 1.93 µg internally), personal items/toys (1.1–12 μg/g or 530–700 µg) and household items such as ironing and cooking implements (0.39–1.0 μg/g or 27–77 µg) as shown in Figure 1. The contents of the vacuum cleaner bag were more significantly elevated (97 μg/g or 68,000 µg) as this reflects the concentration of methamphetamine in dust/fragments regularly vacuumed from the floor surfaces at the property as shown in Figure 1.

Some samples could not be analysed as bulk samples and as a result, the laboratory collected a wipe sample from the surface of these items. This analysis does not indicate the mass of contamination that may be present throughout the material, rather it reflects the mass that may be present at the surface and can be recovered using a surface wipe. For these items, the levels reported on the toothbrushes, laundry roof hatch in the ceiling (not made from plasterboard) and the hallway timber frame exceeded the ACC Guideline of 0.5 µg/100 cm^2^. There are some personal items and tiles where the surface residue level of methamphetamine is below the ACC Guideline. Analysis of what may have adsorbed into some of the porous items such as the wooden items, however, has not been undertaken and remains unknown.

## 4. Discussion

These data indicate that the methamphetamine contamination present in the property was, and likely remains, highly transferable and mobile, and has contaminated all the items brought into the property post-manufacture that were tested. It is also clear that the levels detected by wipe sampling of wall surfaces are not indicative of the level of contamination within or on other surfaces and objects.

Contamination of homes with methamphetamine during the cooking of the drug is well documented [14,17,18]. Our data demonstrate that methamphetamine has continued to be mobilised in a home post-manufacture as the property has been under new ownership for a period exceeding five years and was occupied for the first two years. This suggests that the methamphetamine is not breaking down or being removed and is constantly being transferred from contaminated to non-contaminated objects. Vacuuming and other household activities might have enhanced the rate of transfer. Notably, the United States Environmental Protection Agency has suggested that “contents brought into a former lab after the cook has vacated should be given special consideration [as] these items are likely to be less contaminated” [11]. This contrasts with advice from Abdullah and Miskelly [19] who have stressed that it is “important to remove … obvious pseudoephedrine or methamphetamine-contaminated surfaces prior to heating, ventilation or sealing of a clandestine laboratory to avoid redistribution of material around the site” [19].

Whilst contamination with methamphetamine during cooking has been well documented, the extent of the transferability of contamination is less well studied. Figure 1 presents the internal contamination of the home during cooking, which was extensive. However, what is of greater concern is the level of contamination of objects that were brought into the home post-cooking (also shown in Figure 1). As outlined in the introduction, the house was sold to new owners and was inhabited for several years before being vacated, and was subsequently left uninhabited for several more years. While it is not possible to determine whether the levels we report on the objects introduced represent stable concentrations (i.e., it is not clear whether they were higher or lower over this period) over the period of habitation and vacancy, it is clear that the transference was high and that contamination is extensive. The sampling occurred between October 2014 and November 2018, which means that there could have been movement of the methamphetamine between surfaces through air movement, volatilisation and, to a small degree, through foot traffic and other transfer mechanisms. However, the house was uninhabited after May 2013 and was locked with the windows closed, so it is assumed that changes in methamphetamine concentrations in any of the materials over this time would be minimal. It is proposed that methamphetamine transfer primarily occurred when the family was living in the house undertaking activities such as vacuuming, moving around, cleaning, etc.

Review of the surface wipe samples collected from within the home between 2014 (prior to the property being vacated by the owners) and 2017–2018, indicate that methamphetamine that can be sampled from the walls remains largely the same. There is no evidence of any degradation or loss of surface residues over this period of time.

The use of a surface wipe sampling method from painted gyprock walls to assess and evaluate methamphetamine contamination in this home does not provide any information about the mass of methamphetamine that may be within the building materials and furnishings in the home which adults and children may be exposed to during occupation. Exposure in a home will include contact with a wide range of materials and surfaces. The analysis data suggests a much more significant mass or reservoir of methamphetamine remains within building materials and furnishings in the property.

Reviewing the data collected from the plasterboard materials (outer paper data is shown in Table 2) indicates that the mass present in the paper (painted surface and paper of the plasterboard) is higher than reported in the surface wipe samples collected as shown in Table 1. Further analysis of the plaster/gyprock material indicates that variable amounts of methamphetamine have penetrated into the central part of the plasterboard. Detectable but lower amounts of methamphetamine remain present within the inner paper of the plasterboard wall. This is the internal surface against the timber frame. Methamphetamine contamination is also present on the timber frame and within the roof insulation materials indicating the contamination has moved into the porous building materials.

The most significant mass (15–150 μg/g or 2200–20,000 µg) of methamphetamine remaining in the property was reported to be within the blinds. These are plastic blinds that were present in the property when manufacture was suspected to have been undertaken. This is consistent with unpublished observations from other properties where higher levels of methamphetamine are present in materials such as PVC, polyurethane and stained/varnished timbers.

Given the mass of methamphetamine contamination that remains in the materials in the home and observing no degradation of methamphetamine in surface wipe samples over time, the significant transferability of this contamination to household items is now better understood. In addition, the previously reported exposure and uptake of methamphetamine by the family living in the property [4] can be more explicitly evaluated. (Note: all family members experienced significant health effects. A detailed description of these effects can be found in Wright, Kenneally, Edwards and Walker [4]). An important next step is for future studies to assess the levels of contamination with methamphetamine in houses and household items and compare these environmental exposures with health outcomes and biomarkers of exposure.

## 5. Conclusions

The data collected from this property raises questions about the adequacy of characterising contamination and making decisions about the extent of remediation required based solely on the use of limited surface wipe samples. It is clear that the mass of methamphetamine present varies significantly between different materials, and actual exposure will likely reflect a mix of potential intakes from a range of these different materials. Remediation is required to be undertaken on these properties to ensure they are safe for future occupants. Without fully understanding the extent of contamination that is present, not only on surfaces but also within the building materials and surfaces/items that people are exposed to on a daily basis, it is difficult to ensure that the correct and most effective remedial approaches are taken.

## Figures and Tables

**Table 1:** Surface wipe samples sampled at different times from different areas (µg/100 cm^2^).

Room ID	31 October 2014	10 March 2017	23 November 2018
Laundry wall	23	35	15
Laundry ceiling hatch		11 *	
Kitchen/family meals wall	14	20	
Living/lounge room wall	12	29	
Hallway wall	26	35	
Bedroom 4 wall		17	
Bedroom 3 wall			17
Bedroom 2 wall			69–110
Bedroom 1 wall			11

* analysed from bulk house samples collected in April 2017.

**Table 2:** Plasterboard results from different rooms (outside/painted paper, internal gyprock/plaster and inside paper) (approximately µg/100 cm^2^).

Room ID	Outside Paper	Internal Gyprock	Inside Paper
Laundry wall	44	3.9	0.34
Kitchen/family meals wall	120	6.3	1.2
Living/lounge wall	72	2.1	0.13
Living/lounge ceiling	25	35	0.64
Hallway wall	320	2	0.5
Hallway ceiling	200	730	22
Bedroom 4 wall	36	8.5	3.3
Bedroom 4 ceiling	35	73	1.8
Bedroom 3	93	7.9	4.3
Bedroom 2	730	130	2.9
Bedroom 1 wall	56	3.2	0.22
Bedroom 1 ensuite wall	96	25	0.15
